# Predictive value of stress-induced hyperglycemia for 28-day all-cause mortality in ICU patients with sepsis: a MIMIC-IV-based retrospective cohort study

**DOI:** 10.1016/j.clinsp.2026.101123

**Published:** 2026-09-19

**Authors:** Jiahui Ma, Xu Wang, Yan Zhang, Chunyan Ge

**Affiliations:** a Department of Critical Care Medicine, Hai'an Hospital Affiliated to Nantong University, Nantong Jiangsu, China; b Department of Nursing, Hai'an Hospital Affiliated to Nantong University, Nantong, Jiangsu, China

**Keywords:** Stress-induced hyperglycemia, Sepsis, Mortality risk, MIMIC-IV

## Abstract

**Background:**

This study explored the link between Stress-Induced Hyperglycemia (SIH) and 28-Day All-Cause Mortality (28D-ACM) in patients with sepsis, focusing on the predictive values of stress hyperglycemia ratios 1 and 2 (SHR1 and SHR2) and Glucose Gap (GG).

**Methods:**

This retrospective cohort study included ICU patients with sepsis from the MIMIC-IV database. The relationships between the three SIH metrics and 28D-ACM risk were analyzed using multivariate Cox proportional hazards models, Kaplan-Meier curves, and restricted cubic spline analysis. The predictive performances of these metrics within a basic model were assessed using the Receiver Operating Characteristic (ROC) curve and Integrated Discrimination Improvement (IDI) index.

**Results:**

The study included 12,582 ICU patients with sepsis, with a 28D-ACM rate of 13.63%. In the fully adjusted model, SHR1, SHR2, and GG were independently associated with 28D-ACM risk, with Hazard Ratios (HR) and 95% Confidence Intervals (95% CIs) of 1.158 (1.029, 1.304), 1.18 (1.046, 1.332), and 1.133 (1.008, 1.275), respectively. Increased SIH metrics correlated positively with higher mortality risk (p_trend_< 0.05). The Kaplan-Meier curve indicated that higher tertiles of SHR1, SHR2, and GG resulted in increased cumulative incidence of 28D-ACM (p < 0.05). Additionally, integrating SHR1 and SHR2 enhanced the model's predictive accuracy for 28D-ACM (p < 0.05).

**Conclusions:**

SHR1, SHR2, and GG are significantly associated with 28D-ACM risk in ICU patients with sepsis. SHR1 showed statistical association with mortality and modest incremental value, but clinical utility for risk stratification is limited and needs external validation.

## Highlights

• SHR1, SHR2, and glucose gap independently predict 28-day mortality in ICU sepsis.
• Higher SIH metrics correlate with increased cumulative mortality incidence.
• Adding SHR1 and SHR2 improves baseline model predictive accuracy.
• SHR1 offers superior prognostic value over other stress hyperglycemia indices.
• Dose-response trend shows rising mortality risk across SIH metric tertiles.

## Introduction

Sepsis is a condition characterized by organ dysfunction resulting from dysregulated host responses to infection^1^ and is a leading cause of death among hospitalized patients worldwide. According to the Global Burden of Diseases, Injuries, and Risk Factors Study (GBD) 2017,^2^ there were 48.9 million cases of sepsis and 11 million sepsis-related deaths worldwide, further emphasizing the importance of identifying sepsis and its risk factors to improve outcomes and prevent the condition.

Stress-Induced Hyperglycemia (SIH) is a protective response to intense (pathological, physiological, and psychological) stress, manifested by a transient increase in blood glucose.^3^ SIH is a common glucose metabolism disorder in critically ill patients, with an incidence of up to 73%.^4^ Stress Hyperglycemia Ratio (SHR)1, SHR2, and Glucose Gap (GG) are emerging metrices for determining glycemic stress,^5^^,^^6^ and they are calculated based on Admission Glucose (AG) and glycated Hemoglobin (HbA1c) levels.^7^

SIH is linked to increased mortality in patients with severe diseases such as acute ischemic stroke,^5^ acute kidney injury,^8^ acute cerebral hemorrhage,^9^ and trauma.^10^ However, whether SIH is associated with mortality risk in Intensive Care Unit (ICU) patients with sepsis is not well understood, and comparative studies of different SIH metrics in predicting outcomes for these patients are currently lacking. The present study aims to investigate the influence and predictive performance of different SIH metrics on 28D-ACM risk in ICU patients with sepsis, thereby providing insights to the management and clinical decision-making for these patients.

## Methods

### Data collection

The subjects for this study originated from the MIMIC-IV,^11^ which is a comprehensive, single-center database comprising de-identified patient-related information maintained by the Laboratory for Computational Physiology at the Massachusetts Institute of Technology. The author of this study (MJH) was granted access to MIMIC-IV (ID: 13254084) and extracted all relevant data. The study was conducted in accordance with the Declaration of Helsinki. The data was approved by the review boards of MIT and Beth Israel Deaconess Medical Center. Since all patient information in MIMIC-IV has been deidentified and is publicly accessible, informed consent was exempted.

### Inclusion criteria

Patients diagnosed with sepsis per the Sepsis 3.0 diagnostic criteria, along with an infection and Sequential Organ Failure Assessment (SOFA) score of ≥ 2. The methodology for retrieving patients with sepsis who met Sepsis 3.0 from MIMIC-IV was consistent with previously published studies.^12^^,^^13^ For patients with multiple ICU admissions, only data from the initial admission were collected.

### Exclusion criteria

(1) Patients not admitted to ICU; (2) Patients with missing HbA1c and AG values in laboratory tests; (3) Age < 18-years.

The procedures for inclusion and exclusion of research subjects are shown in Fig. 1.

**Fig. 1:**
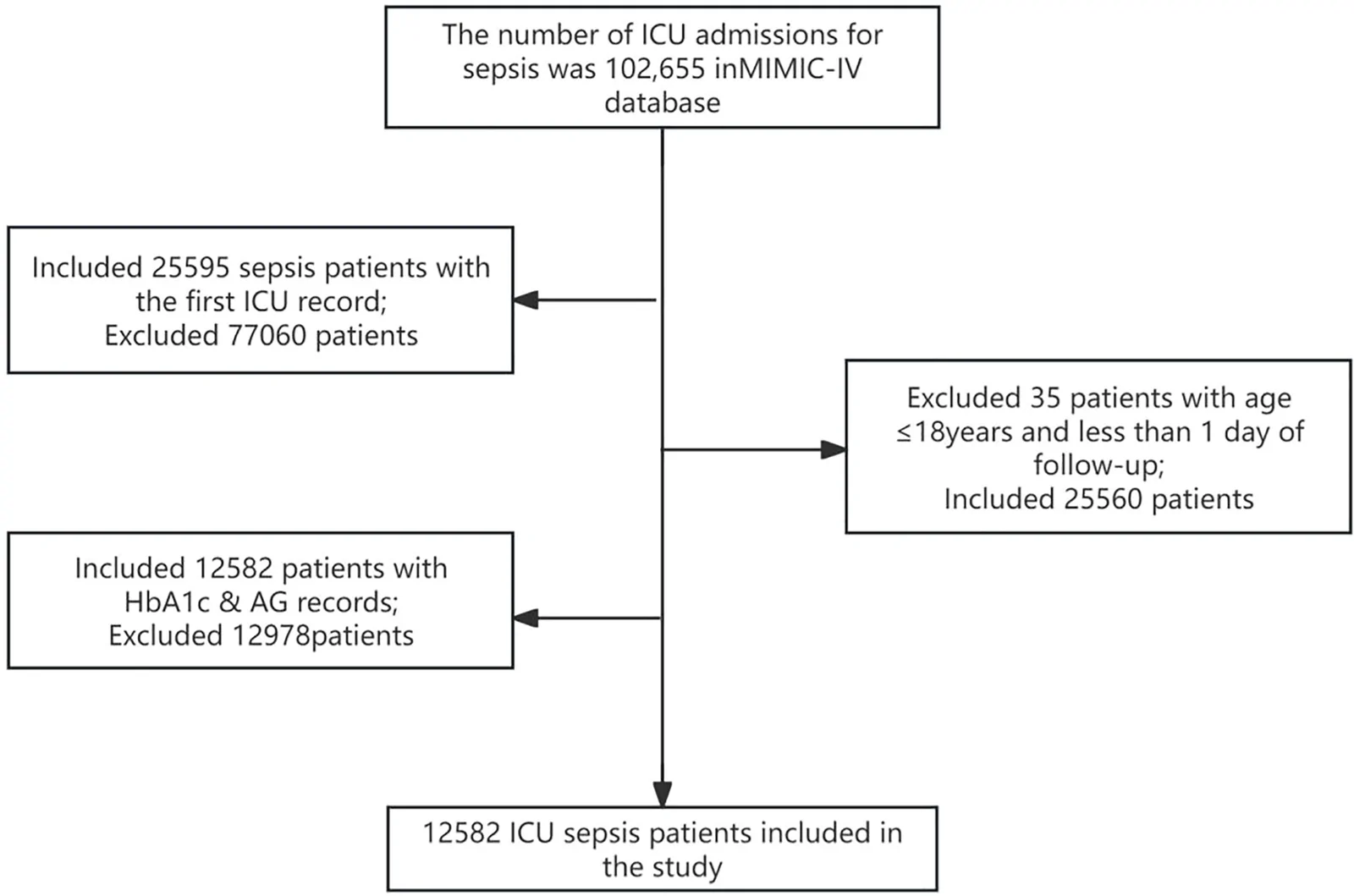
Flow chart of inclusion of study population.

### Data extraction

Demographics, comorbidities, laboratory parameters, and clinical outcomes were extracted from MIMIC-IV using Structured Query Language (SQL). Demographics included sex, age, and ethnicity; laboratory parameters included Platelet Count (PLT), Total Bilirubin (TBIL), Albumin (ALB), Blood Urea Nitrogen (BUN), Calcium (Ca), Chloride (Cl), Creatinine (Cr), Glucose (GLU), Glycated Hemoglobin (HbA1c), Hemoglobin (HGB), Potassium (K), Red Blood Cells (RBCs), Sodium (Na), and White Blood Cells (WBCs); comorbidities included atrial fibrillation, cerebrovascular disease, chronic liver disease, congestive heart failure, Chronic Pulmonary Disease (COPD), diabetes, Human Immunodeficiency Virus (HIV), hypertension, malignant tumor, and myocardial infarction. All of the above parameters were measured within 24-hours of ICU admission. For variables that were measured multiple times, only the first measurements were used. The start date of follow-up was the date of ICU admission, and the primary outcome was 28D-ACM. To minimize reverse causality bias, data collected after an outcome event was considered invalid. Missing laboratory values are common in MIMIC-IV. To minimize bias due to sample exclusion, only variables with ≤10% missing values were imputed by multiple imputation.

The three SIH metrics were calculated as follows: SHR1 (mmoL/L) = AG/[(1.59 × HbA1c) - 2.59],^7^ SHR2 = AG/HbA1c,^14^ GG (mmol/L) = AG-[(1.59 × HbA1c) - 2.59].^6^ The SIH metrics were categorized into tertiles, using the lowest tertile (Q1) as the reference for each SIH metric.

### Statistical analysis

Statistical analysis was performed using R 4.4.0. Continuous variables are expressed as mean (Standard Deviation [SD]) or median (Interquartile Range [IQR]) and compared using the *t*-test or non-parametric tests. Categorical variables are presented as frequency and percentage (%) and compared using the Chi-Square test. The correlation of SHR1, SHR2, and GG with mortality was evaluated based on the Hazard Ratio (HR) and its 95% Confidence Interval (95% CI) using the Cox hazard regression model. Adjustments were made on several confounders (Model 1: Unadjusted; Model 2: Adjusted for age, race, sex, PLT, TBIL, ALB, BUN, Ca, Cl, Cr, K, RBCs, Na, and WBCs; Model 3: Adjusted for atrial fibrillation, cerebrovascular disease, chronic liver disease, congestive heart failure, COPD, diabetes, HIV, hypertension, malignant tumor, and myocardial infarction). The potential non-linear relationships between SHR1, SHR2, GG and patient outcomes were determined using Restricted Cubic Splines (RCS). Survival was compared between subgroups of SHR1, SHR2, and GG using the Kaplan-Meier curves. The Area Under the ROC Curve (AUC) and Integrated Discriminant Improvement (IDI) were calculated to evaluate the improvement in predictive performance of the basic model after incorporating SHR1, SHR2, and GG. Of note, no internal (e.g., bootstrap resampling or cross-validation) or external validation was performed, which should be considered when interpreting predictive performance. A two-sided p < 0.05 was considered statistically significant.

## Results

### Baseline characteristics

A total of 12,582 eligible ICU patients with sepsis were selected from the MIMIC-IV. The patients were divided into the surviving group (n = 10,867) and non-surviving group (n = 1,715) based on their 28D-ACM, and the 28D-ACM rate was 13.63%. Non-survivors were older than survivors. Additionally, patients with atrial fibrillation, cerebrovascular disease, chronic liver disease, diabetes, hypertension, and myocardial infarction had higher 28D-ACM rates in the non-surviving group than in the surviving group. Non-survivors showed significantly higher levels of TBIL, ALB, BUN, Ca, Cl, Cr, GLU, HGB, K, RBC count, and WBC count (p < 0.05) (Table 1).

**Table 1:** Basic characteristics of the patients.

**Variables**	**Total (n = 12582)**	**Survival (n = 10867)**	**Death (n = 1715)**	**p**
Age/year (median [IQR])	66.00 [56.00, 76.00]	66.00 [56.00, 75.00]	71.00 [60.00, 80.00]	<0.001
Male, n (%)	7662 (60.9)	6694 (61.6)	968 (56.4)	<0.001
Race (%)				<0.001
Asian	211 (1.7)	184 (1.7)	27 (1.6)	
Black	1409 (11.2)	1179 (10.8)	230 (13.4)	
White	8359 (66.4)	7323 (67.4)	1036 (60.4)	
Other	2603 (20.7)	2181 (20.1)	422 (24.6)	
Lab indexes (median [IQR])				
Blood platelets/(K/uL)	223.00 [175.00, 280.00]	223.00 [176.00, 280.00]	224.00 [168.00, 282.00]	0.357
Total bilirubin/(mg/dL)	0.50 [0.30, 0.80]	0.50 [0.30, 0.80]	0.50 [0.30, 0.90]	<0.001
Albumin/(g/dL)	3.90 [3.40, 4.30]	3.90 [3.50, 4.30]	3.70 [3.10, 4.20]	<0.001
Blood urea nitrogen/(mg/dL)	19.00 [14.00, 27.00]	19.00 [14.00, 26.00]	21.00 [15.00, 32.00]	<0.001
Calcium/(mg/dL)	8.90 [8.30, 9.30]	8.90 [8.30, 9.30]	8.90 [8.40, 9.40]	0.001
Chloride/(mEq/L)	102.00 [100.00, 105.00]	102.00 [100.00, 105.00]	102.00 [99.00, 105.00]	0.001
Creatinine/(mg/dL)	1.00 [0.80, 1.30]	1.00 [0.80, 1.30]	1.10 [0.80, 1.50]	<0.001
Glucose/(mg/dL)	117.00 [96.00, 158.00]	117.00 [96.00, 156.00]	122.00 [98.00, 173.50]	<0.001
HbA1c/%	5.90 [5.50, 6.70]	5.90 [5.50, 6.70]	6.00 [5.50, 6.80]	0.782
Hemoglobin/(g/dL)	12.80 [11.40, 14.10]	12.90 [11.50, 14.10]	12.40 [10.90, 13.80]	<0.001
Potassium/(mEq/L)	4.20 [3.90, 4.60]	4.20 [3.90, 4.50]	4.30 [3.90, 4.70]	<0.001
Red blood cells/(m/uL)	4.25 [3.79, 4.67]	4.27 [3.82, 4.68]	4.11 [3.64, 4.59]	<0.001
Sodium/(mEq/L)	139.00 [137.00, 141.00]	139.00 [137.00, 141.00]	139.00 [136.00, 142.00]	0.293
White blood cells/(K/uL)	7.90 [6.20, 10.50]	7.90 [6.20, 10.40]	8.50 [6.40, 12.00]	<0.001
Complications, n (%)				
Atrial Fibrillation	5225 (41.5)	4472 (41.2)	753 (43.9)	0.034
Cerebrovascular disease	523 (4.2)	435 (4.0)	88 (5.1)	0.035
Chronic Liver Disease	315 (2.5)	286 (2.6)	29 (1.7)	0.025
Congestive Heart Failure	2618 (20.8)	2254 (20.7)	364 (21.2)	0.67
COPD	1286 (10.2)	1108 (10.2)	178 (10.4)	0.85
Diabetes	5890 (46.8)	5047 (46.4)	843 (49.2)	0.039
HIV	142 (1.1)	119 (1.1)	23 (1.3)	0.439
Hypertension	6379 (50.7)	5599 (51.5)	780 (45.5)	<0.001
Malignant tumor	178 (1.4)	150 (1.4)	28 (1.6)	0.476
Myocardial infarction	2583 (20.5)	2128 (19.6)	455 (26.5)	<0.001
SIH-related index (median [IQR])				
SHR1	0.95 [0.80, 1.19]	0.94 [0.80, 1.18]	1.00 [0.81, 1.31]	<0.001
SHR2	1.09 [0.92, 1.39]	1.08 [0.92, 1.37]	1.15 [0.94, 1.54]	<0.001
GG	-0.35 [-1.41, 1.35]	-0.38 [-1.42, 1.25]	-0.01 [-1.36, 2.27]	<0.001

HbA1c, Glycated Hemoglobin; CPOD, Chronic Obstructive Pulmonary Disease; HIV, Human Immunodeficiency Virus; SIH, Stress-Induced Hyperglycemia; SHR, Stress Hyperglycemia Ratio; GG, Glucose Gap.

### Correlation analysis of SHR1, SHR2 and GG with 28D-ACM risk in ICU patients with sepsis

Separate incorporation of SHR1, SHR2 and GG into the Cox regression for Model 1 resulted in significantly higher 28D-ACM risk in the Q3 group than in the Q1 group (HR_SHR1_ = 1.351, HR_SHR2_ = 1.375, HR_GG_ = 1.314, p < 0.05). Model 2 was constructed based on Model 1, with adjustments for demographics and laboratory parameters. Analysis of Model 2 demonstrated that the SIH metrics were correlated with mortality risk in both the Q2 and Q3 groups, and mortality was significantly increased in the Q3 group compared to the Q1 group (HR_SHR1_ = 1.180, HR_SHR2_ = 1.188, HR_GG_ = 1.156, p < 0.05). Model 3 (Model 2 with adjustments for comorbidities) also revealed significant differences in mortality risk between the Q3 and Q1 groups (HR_SHR1_ = 1.158, HR_SHR2_ = 1.18, HR_GG_ = 1.133, p < 0.05). Moreover, the mortality risk of ICU patients with sepsis increased as the SIH metrics increased (p_trend_ < 0.05) (Table 2).

**Table 2:** Correlation of SHR1, SHR2, and GG metrics with 28-day mortality risk in ICU patients with sepsis.

**Variables**	**Model 1**			**Model 2**			**Model 3**		
	**HR (95% CI)**	**p**	**p-trend**	**HR (95% CI)**	**p**	**p-trend**	**HR (95% CI)**	**p**	**p-trend**
SHR1 tertile			<0.001			0.004			0.011
Q1 (≤ 0.85)	Reference			Reference			Reference		
Q2 (> 0.85, ≤ 1.09)	0.952 (0.843, 1.077)	0.435		0.940 (0.831, 1.064)	0.328		0.925 (0.817, 1.047)	0.216	
Q3 (> 1.09)	1.351 (1.206, 1.514)	<0.001		1.180 (1.049, 1.327)	0.006		1.158 (1.029, 1.304)	0.015	
SHR2 tertile			<0.001			0.004			0.007
Q1 (≤ 0.98)	Reference			Reference			Reference		
Q2 (> 0.98, ≤ 1.26)	1.053 (0.932, 1.189)	0.41		1.026 (0.908, 1.160)	0.681		1.017 (0.899, 1.149)	0.792	
Q3 (> 1.26)	1.375 (1.226, 1.544)	<0.001		1.188 (1.054, 1.338)	0.005		1.18 (1.046, 1.332)	0.007	
GG tertile			<0.001			0.011			0.029
Q1(≤ -1.05)	Reference			Reference			Reference		
Q2 (> -1.05, ≤ 0.62)	0.918 (0.812, 1.038)	0.171		0.920 (0.813, 1.040)	0.183		0.896 (0.791, 1.015)	0.085	
Q3 (> 0.62)	1.314 (1.174, 1.472)	<0.001		1.156 (1.028, 1.299)	0.015		1.133 (1.008, 1.275)	0.037	

Model 1, Crude model; Model 2, adjusted for Model 1 + age, race, gender, blood platelets, total bilirubin, albumin, blood urea nitrogen, calcium, chlorid, creatinine, potassium, red blood cells, sodium, white blood cells; Model 3, adjusted for Model 2 + Atrial Fibrillation, cerebrovascular disease, Chronic Liver Disease, Congestive Heart Failure, COPD, diabetes, HIV, Hypertension, Malignant tumor, myocardial infarction.HR, Hazard Ratio; CI, Confidence Interval; SHR, Stress Hyperglycemia Ratio; GG, Glucose Gap; HIV, Human Immunodeficiency Virus; CPOD, Chronic Obstructive Pulmonary Disease.

RCS analysis showed that SHR1 was linearly and positively correlated with mortality risk in ICU patients with sepsis (p_overall_ < 0.05, p_non-linear_ > 0.05) (Fig. 2A). Similarly, the risk of 28D-ACM increased linearly as SHR2 and GG increased (p_overall_ < 0.05, p_non-linear_ > 0.05) (Fig. 2B‒C).

**Fig. 2:**
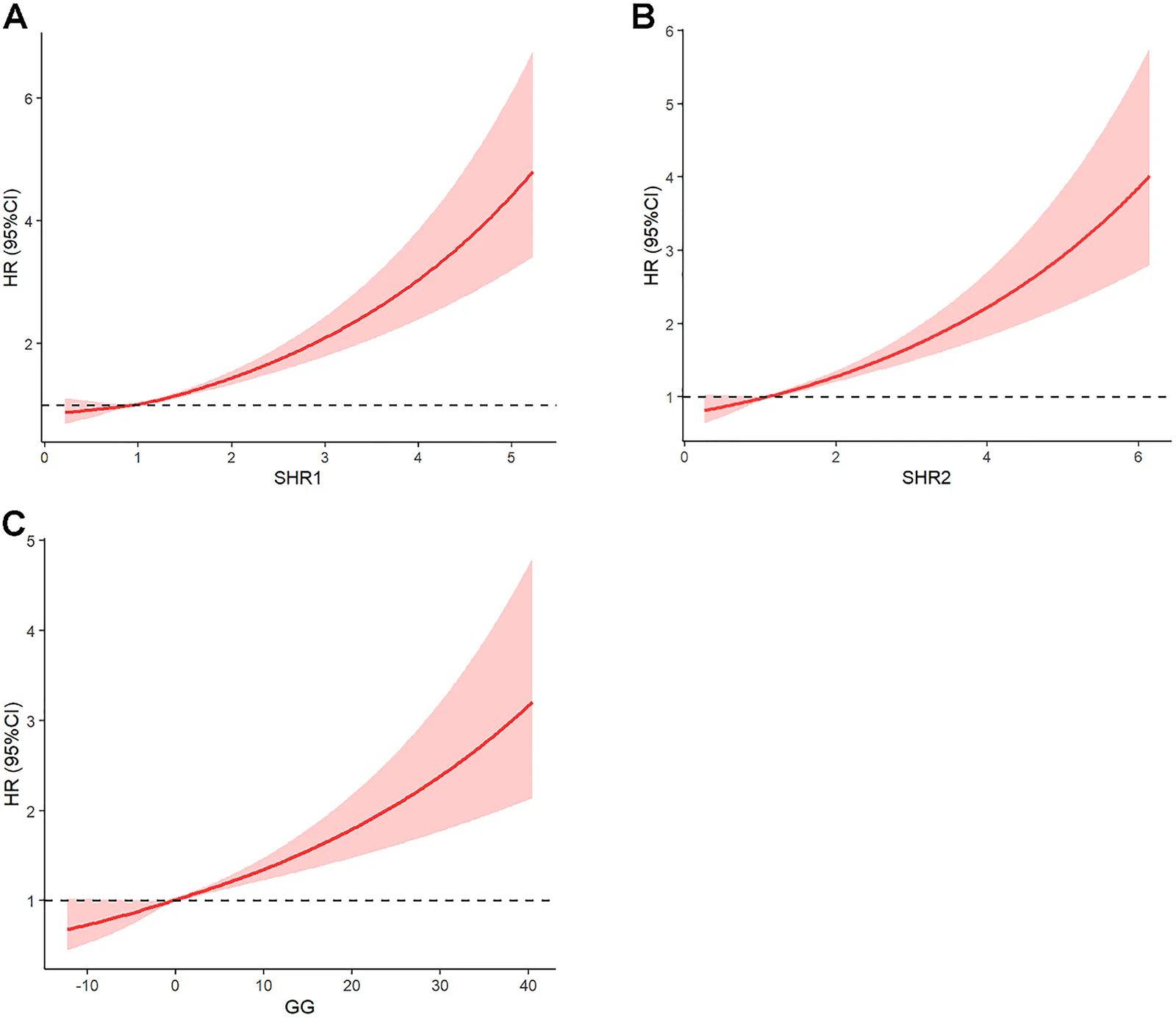
(A) SIH-restricted cubic spline plot -SHR1, (B) SIH-restricted cubic spline plot -SHR2, (C) SIH-restricted cubic spline plot -GG.

Survival analysis using the Kaplan-Meier curve demonstrated that the SHR1 Q3 group had significantly shorter survival compared to the SHR1 Q1 and Q2 groups (p < 0.05) (Fig. 3A). Likewise, the SHR2 and GG Q3 groups had the lowest 28-day survival rates compared to their respective Q1 and Q2 groups (p < 0.05) (Fig. 3B‒C).

**Fig. 3:**
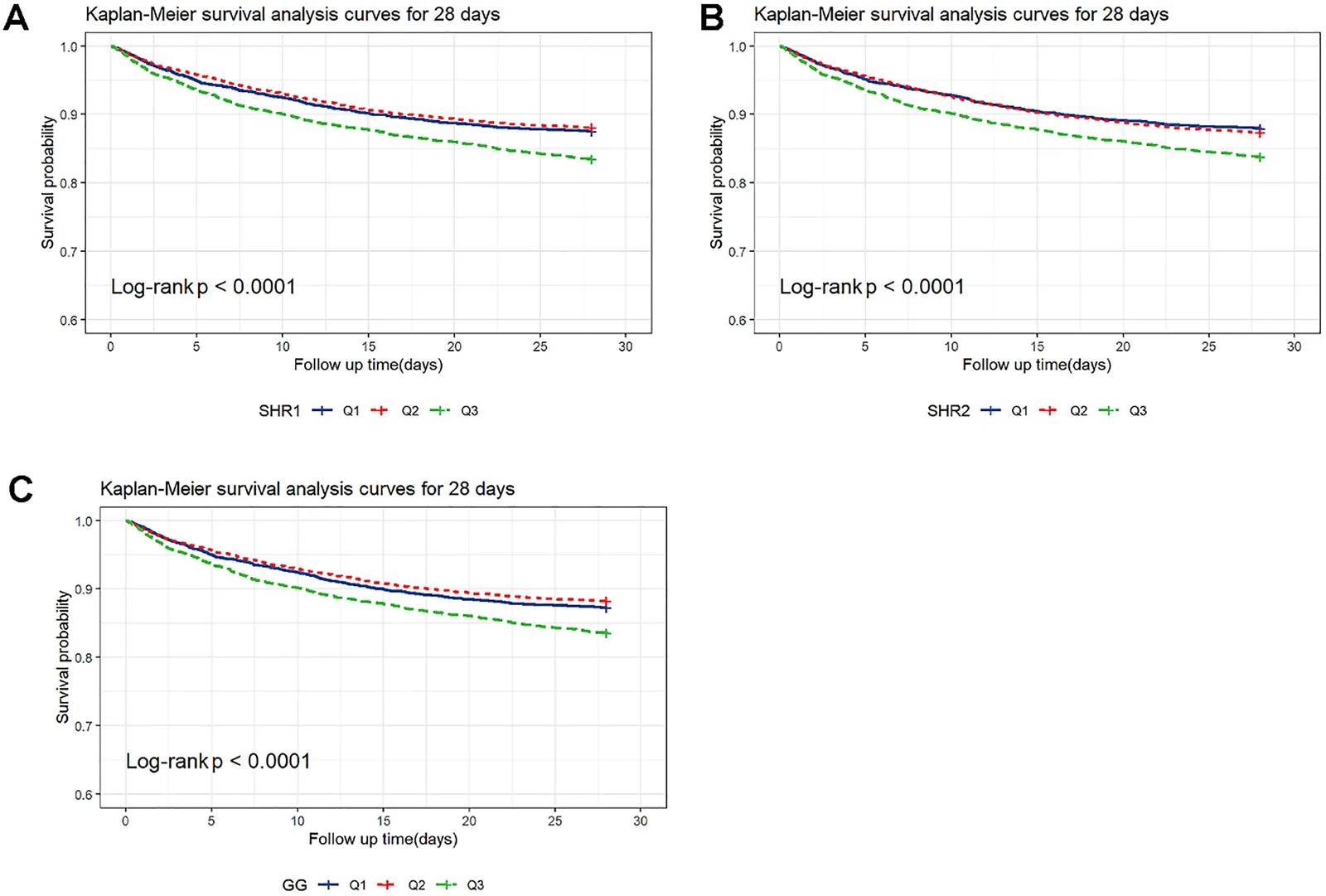
(A) Kaplan-Meier curves for grouping SIH indicators -SHR1, (B) Kaplan-Meier curves for grouping SIH indicators -SHR2, (C) Kaplan-Meier curves for grouping SIH indicators -GG.

### Predictive performance and incremental effect of SHR1, SHR2 and GG

Next, the incremental effects of SHR1, SHR2, and GG on the predictive performance of the basic model (including PLT, TBIL, ALB, BUN, Ca, Cl, Cr, K, RBCs, age, Na, WBCs, atrial fibrillation, cerebrovascular disease, chronic liver disease, congestive heart failure, COPD, diabetes, HIV, hypertension, malignant tumor, myocardial infarction, race, and gender) were evaluated. The AUCs for comparison and p-values are provided in Table 3. The DeLong test revealed that SHR1 and SHR2, but not GG, improved the prediction of mortality risk of the basic model (AUCbase+SHR1 [0.673 (0.660‒0.687)] vs. AUCbase+SHR2 [0.672 (0.659‒0.686)], p < 0.05). Therefore, the IDI was calculated to assess the impact of each SIH metric on the mortality risk of ICU patients with sepsis. IDI is a metric for assessing improvements in the predictive performance of a model. A positive IDI value indicates an enhancement in predictive accuracy, while a negative IDI value suggests a decrease in predictive performance.^15^ The results showed that the SHR1, SHR2, and GG risk models had significantly enhanced predictive performance compared to the risk model without SIH metrics (IDIbase+SHR1 [0.0022 (0.001‒0.0033)] vs. IDIbase+SHR2 [0.0015 (0.0005‒0.0025)] vs. IDIbase+GG [0.0006 (0‒0.0013)], p < 0.05). Collectively, these data demonstrated that the incorporation of SHR1 significantly improved the prediction of 28D-ACM by the basic model compared to SHR2 and GG (p < 0.05).

**Table 3:** Improvement in 28-day mortality risk in ICU septic patients with increased SHR1, SHR2, and GG metrics.

	**AUC [95% CI]**	**p**^a^	**IDI [95%CI]**	**p**^a^
**Base model**	0.669 [0.655‒0.683]			
**+SHR1**	0.673 [0.660‒0.687]	0.007	0.0022 [0.001‒0.0033]	0.00034
**+SHR2**	0.672 [0.659‒0.686]	0.018	0.0015 [0.0005‒0.0025]	0.00274
**+GG**	0.671 [0.657‒0.684]	0.077	0.0006 [0‒0.0013]	0.04598

a Comparison with the basic model. Basic models include blood platelets, total bilirubin, albumin, blood urea nitrogen, calcium, chlorid, creatinine, potassium, red blood cells, age, sodium, white blood cells, Atrial Fibrillation, cerebrovascular disease, Chronic Liver Disease, Congestive Heart Failure, COPD, diabetes, HIV, Hypertension, Malignant tumor, myocardial infarction, race, gender.

## Discussion

This study examined the effects of SHR1, SHR2, and GG on 28D-ACM risk in ICU patients with sepsis. SHR1, SHR2, and GG were found to be linearly and positively correlated with 28D-ACM risk in these patients. Except for GG, both SHR1 and SHR2 moderately enhanced the predictive performance of the basic risk model for 28D-ACM risk. Although the improvement in IDI was statistically significant, the magnitude was small and its incremental predictive value for clinical correlation warrants further validation in large-cohort studies or other clinical scenarios.

Persistent, severe SIH can compromise immune function and increase the risks of poor prognosis and mortality.^15^ A prospective multicenter cohort study^16^ involving 7662 patients with acute coronary syndrome and a median follow-up of 2.1-years found that high SHR was significantly linked to higher risks of adverse cardiovascular events (HR = 1.53, 95% CI 1.24‒1.88), ACM (HR = 1.80, 95% CI 1.29‒2.51), and unplanned revascularization (HR=1.44, 95% CI 1.09‒1.91) (all p < 0.05). Since cortisol and catecholamines are associated with the severity of injury, SIH often occurs in critically ill trauma patients. Chang et al.^17^ found that among critically ill trauma patients, mortality was 2.13-times more likely in SIH patients compared to diabetic patients after adjusting for sex, age, previous comorbidities, and injury severity scores. Zhang et al.^18^ investigated the impact of SIH on the incidence of complications and functional outcomes, and reported that SIH was a significant independent risk factor for symptomatic vasospasm, delayed cerebral infarction, hydrocephalus, and poor outcome at 12-months in patients with nontraumatic nonaneurysmal subarachnoid hemorrhage.

There is currently no consensus on the impact of SIH on mortality in ICU patients with sepsis. Previous studies have suggested that SIH plays a protective role in reducing mortality in patients with sepsis.^19^^,^^20^ However, the small sample size of these two studies may affect the robustness of the conclusions. In contrast, a large retrospective study of 2312 critically ill patients with sepsis showed that SHR was associated with 28D-ACM and in-hospital mortality in critically ill sepsis patients^21^ Li et al.^22^ examined the association between SHR and 1-year mortality in patients with sepsis and showed that SHR1 values were associated with increased risk of poor prognosis. However, comparative studies of different SIH metrics in predicting the outcomes of ICU patients with sepsis are lacking.

Several studies have assessed the predictive performance of various SIH metrics in disease outcomes. A retrospective study reported that SHR provided better prognostic insights for assessing the relationship between SIH and ischemic stroke outcomes compared to GG.^23^ A study^7^ involving 5249 ICU patients showed that the AUCs were markedly higher for the SHR2 and GG models than for the SHR1 model (AUC_SHR2_ = 0.691 (95% CI: 0.661‒0.720), AUC_GG_ = 0.685 (95% CI: 0.655‒0.714), AUC_SHR1_ = 0.680 (95% CI: 0.650‒0.709), p < 0.05), indicating that SHR2 and GG were superior to SHR1 in predicting 28D-ACM risk in ICU patients. However, there are few studies on the use of different SIH indicators to predict the outcomes of ICU sepsis patients. Therefore, this study compared the three SIH metrics SHR1, SHR2 and GG to reveal the predictive performance of each metric for 28D-ACM in ICU patients with sepsis.

Our study demonstrated a significant correlation between SIH and poor outcomes in ICU patients with sepsis, potentially attributable to several underlying mechanisms. First, SIH promotes the synthesis and release of proinflammatory cytokines,^24^ which have been reported to correlate with poor outcomes in sepsis patients.^25^ Therefore, a lower SIH metric value may have an anti-inflammatory effect and contribute to a reduction in adverse events.^21^ Second, Disseminated Intravascular Coagulation (DIC) is another key contributor to increased mortality from sepsis.^26^ Acute elevation of blood glucose can promote intravascular coagulation abnormalities, leading to sepsis-associated DIC.^27^ Third, inflammation induced by glucose excursions increases ACM risk in sepsis patients.^28^

This study has several highlights. First, the inclusion criteria were determined using the Sepsis 3.0 criteria, which is more in line with the latest definition of sepsis. MIMIC-IV is a large database that contains patient information from the same ICU between 2008 and 2019. Second, IDI analysis demonstrated that adding SHR1, SHR2 and GG to the basic risk model significantly improved the prediction of 28D-ACM risk in ICU patients with sepsis compared to the model without these SIH metrics (p < 0.05). Additionally, this study showed that SHR1 and SHR2 had better predictive performance than GG, which was consistent with previous findings.^21^^,^^22^ Third, more variables were adjusted to ensure the stability of the study results. Last, our results may help physicians evaluate the outcomes of ICU sepsis patients since HbA1c and AG are readily available parameters in clinical practice.

Nevertheless, several limitations should be considered. First, this study may be subject to selection bias, which could hinder the applicability of the findings to other countries and populations. Second, since the prognosis of sepsis may be influenced by immune status, the use of chemotherapeutic agents or immunosuppressants could be a significant confounder. However, the database does not include information on these variables. Third, the absence of model validation (including internal and external validation) limits the generalizability and robustness of the predictive findings. Fourth, important confounders such as illness severity indicators (e.g., lactate levels, vasopressor use, mechanical ventilation) and glycemic management strategies were not available, which may have introduced residual confounding. Fifth, based on the definitions of the three SIH metrics (SHR1, SHR2 and GG), the exclusion of patients without HbA1c and AG measurements may have introduced selection bias towards ICU patients with more stable conditions, potentially limiting generalizability to less-monitored or more acute populations. Sixth, it is worth noting that no internal (e.g., bootstrap resampling or cross-validation) or external validation was performed, which should be considered when interpreting predictive performance. Last, since this is a single-center retrospective study without external validation, bias could not be eliminated. Therefore, a prospective cohort study is warranted to further validate the findings.

## Conclusion

SHR1, SHR2, and GG are significantly associated with 28D-ACM risk in ICU sepsis patients. SHR1 was statistically associated with mortality and showed a modest incremental predictive value; however, its clinical utility for risk stratification remains limited and requires external validation. SIH assessment may be valuable for the early identification of ICU patients with sepsis at high risk of mortality and may contribute to hypothesis generation and requires prospective validation before clinical application.

## Ethics approval and consent to participate

The study was conducted in accordance with the Declaration of Helsinki. The data was approved by the review boards of MIT and Beth Israel Deaconess Medical Center. Because all patient information in the MIMIC database has been deidentified and is publicly accessible, Informed consent was exempted by MIT and Beth Israel Deaconess Medical Center.

## Data availability statement

The author of this study (MJH) was granted access to MIMIC-IV (ID: 13254084) and extracted all relevant data.

## Consent for publication

Not applicable.

## Clinical trial registration

Not applicable.

## Declaration of generative AI

Not applicable.

## Authors’ contributions

All authors contributed to the study conception and design. Writing-original draft preparation: Jiahui Ma, Xu Wang, Yan Zhang, Chunyan Ge; Writing-review and editing: Jiahui Ma; Conceptualization: Jiahui Ma; Methodology: Xu Wang; Formal analysis and investigation: Xu Wang, Yan Zhang; Resources: Chunyan Ge; Supervision: Chunyan Ge, and all authors commented on previous versions of the manuscript. All authors read and approved the final manuscript.

## Funding

This work was supported by Hai 'an People's Hospital “Based on the dialectical the nursing of patients with Chronic Kidney Disease (CKD) with type 2 diabetes symptoms group management scheme and implementation” (n°202449), Research Project of Nantong Municipal Health Commission-Youth Directive Project “Construction and effect evaluation of WeChat platform for precision nursing of type 2 diabetes based on symptom management theory” (QN2023052) and supported by Nantong University Special Research Fund for Clinical Medicine: “Research on the Intervention Effect of Syndrome Differentiation and Nursing Care on Symptom Management in Patients with Type 2 Diabetes Mellitus Complicated with Chronic Kidney Disease” (Grant n° 2025HQ001).

## Conflicts of interest

The authors declare no conflicts of interest.
